# Expression of pair rule gene orthologs in the blastoderm of a myriapod: evidence for pair rule-like mechanisms?

**DOI:** 10.1186/1471-213X-12-15

**Published:** 2012-05-17

**Authors:** Ralf Janssen, Wim G M Damen, Graham E Budd

**Affiliations:** 1 Department of Earth Sciences, Uppsala University, Villavägen 16, 752 36, Uppsala, Sweden; 2 Department of Genetics, Friedrich-Schiller-University Jena, Philosophenweg 12, 07743, Jena, Germany

**Keywords:** Evolution, Pair rule patterning, Segmentation, *Paired*, *Even-skipped*, *Runt*, *Hairy*, *Odd-paired*, *Sloppy-paired*, *Odd-skipped*

## Abstract

**Background:**

A hallmark of *Drosophila* segmentation is the stepwise subdivision of the body into smaller and smaller units, and finally into the segments. This is achieved by the function of the well-understood segmentation gene cascade. The first molecular sign of a segmented body appears with the action of the pair rule genes, which are expressed as transversal stripes in alternating segments. *Drosophila* development, however, is derived, and in most other arthropods only the anterior body is patterned (almost) simultaneously from a pre-existing field of cells; posterior segments are added sequentially from a posterior segment addition zone. A long-standing question is to what extent segmentation mechanisms known from *Drosophila* may be conserved in short-germ arthropods. Despite the derived developmental modes, it appears more likely that conserved mechanisms can be found in anterior patterning.

**Results:**

Expression analysis of pair rule gene orthologs in the blastoderm of the pill millipede *Glomeris marginata* (Myriapoda: Diplopoda) suggests that these genes are generally involved in segmenting the anterior embryo. We find that the *Glomeris pairberry-1* ( *pby-1*) gene is expressed in a pair rule pattern that is also found in insects and a chelicerate, the mite *Tetraynchus urticae*. Other *Glomeris* pair rule gene orthologs are expressed in double segment wide domains in the blastoderm, which at subsequent stages split into two stripes in adjacent segments.

**Conclusions:**

The expression patterns of the millipede pair rule gene orthologs resemble pair rule patterning in *Drosophila* and other insects, and thus represent evidence for the presence of an ancestral pair rule-like mechanism in myriapods. We discuss the possibilities that blastoderm patterning may be conserved in long-germ and short-germ arthropods, and that a posterior double segmental mechanism may be present in short-germ arthropods.

## Background

In *Drosophila*, a hierarchic segmentation gene cascade acts to stepwise pattern the early embryo into single segments (reviewed in [1-3]). Maternally provided factors such as *bicoid* and *hunchback*, rest at the top of this hierarchy, which allows these genes to regulate zygotically expressed gap genes (GGs) ([4], reviewed in [5]). The GGs, that are expressed in broad overlapping domains along the anterior-posterior axis of the embryo, regulate the pair rule genes (PRGs) in transversal stripes in alternating segment primordia [6]. During a subsequent phase of segment formation, the PRGs are often expressed in a single segmental periodicity and, at this point, act as segment-polarity genes (SPGs) (e.g. [7,8]). In a combinatorial mode the PRGs regulate the expression of the SPGs, which maintain the parasegment boundaries and define the segments’ polarity.

This mode of segment formation is called long-germ developmental mode because all segments are patterned from a pre-existing field of cells, the blastoderm (e.g. [9]). *Drosophila* development, however, is derived, and is, at best, comparable to some groups of higher insects. Only the most anterior segments form from the blastoderm in the majority of arthropods, while the posterior segments are added in a single or double segment period from a posterior segment addition zone (SAZ) [10]. This ancestral mode of development and segment formation is called short-germ developmental mode.

Recent studies have shown that the mechanisms and gene interactions acting at the bottom level of the *Drosophila* segmentation gene cascade, i.e. SPGs and Hox genes, appear to be highly conserved among arthropods (e.g. [11-15]) and onychophorans [16,17]. At the level of maternally provided effect genes and GGs, however, the segmentation gene hierarchy appears to be less conserved (e.g. [18]). The level at which the PRGs act is intermediate between that of the SPGs and Hox genes, and that of maternally provided effect genes and GGs (e.g. [2]). Examination of PRG expression and function in insects other than *Drosophila* revealed that this level of the segmentation gene cascade is, to some degree, conserved in insects (e.g. [19-21]). The expression profile of PRGs in most insects is, however, somewhat different from that in *Drosophila*. In non-Drosophilid long-germ insects, PRGs are often initially expressed in double-segment wide stripes that later split into a single segmental pattern (e.g. [22,23]). In short-germ insects a similar pattern is found in the anterior blastoderm, but during posterior segment addition PRGs are, like in non-insect arthropods, usually expressed in both dynamic patterns in the SAZ and in stripes in the newly formed segment(s) (e.g. [14,24-28]). It is therefore debatable whether they are involved in a pair rule-like mechanism (e.g. [2,18,29]). Data on early PRG expression or function in the blastoderm in non-insect arthropods are scant [30,31] and for that reason, it is unclear whether a pair rule-like mechanism may be present in anterior patterning.

To shed light on this topic we examined the expression of most of the known *Drosophila* PRG orthologs in the blastoderm of the pill millipede *Glomeris marginata* (Myriapoda). The orthologs of two *Drosophila* PRG genes are not subject of this study: The *fushi-tarazu* gene acts as a classical PRG in *Drosophila*, but in basal hexapods and other arthropods, including *Glomeris*, it may have retained its ancestral role as Hox gene and does not act as a PRG [25,32-34]. The *tenascin-major* ( *ten-m*) gene (aka *odz*) is a rather atypical PRG in *Drosophila*. It does not encode a transcription factor, like all other PRGs, and only has been student in *Drosophila* where it is only expressed in a pair rule pattern on protein level, but not on mRNA level. Therefore we decided not to include *ten-m* in the present analysis. We find that all investigated PRG orthologs, except one, are expressed in transversal stripes that are typical for segmentation genes and which are in patterns that may be in accord with an underlying pair rule-like mechanism. The blastodermal expression of the PRGs is different from that in segments added from the SAZ in *Glomeris*[24]: they do not appear in a strict anterior to posterior order and are often initially expressed in double (or multiple) segment-wide domains.

## Methods

### Species husbandry, gene cloning, in situ hybridization, nuclei staining and documentation techniques

The handling of *Glomeris marginata* is described in [11]. After oviposition, embryos were allowed to develop at room temperature. Staging was done afterwards [11]. The developmental stage of all embryos was determined by using the dye, DAPI (4'-6-Diamidino-2-phenylindole).

Cloning and sequence analysis of the *Glomeris* pair rule gene orthologs has been described in [24].

Single whole mount in situ hybridization was performed as described in [35]. Double whole mount in situ hybridization was performed as described in [12].

Embryos were analyzed under a Leica dissection microscope equipped with either an Axiocam (Zeiss) or a Leica DC100 digital camera. Brightness, contrast, and color values were corrected in all images using the image processing software Adobe Photoshop CS2 (Version 9.0.1 for Apple Macintosh).

## Results

### Morphology of the early *Glomeris* embryo and technical limitations of in situ hybridization experiments

**Figure 1:**
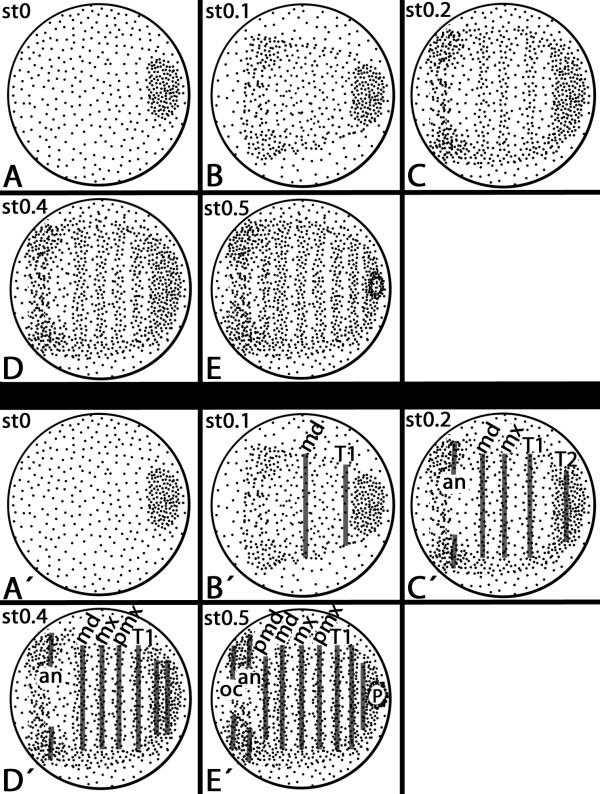
**Schematic drawings representing the early development of *Glomeris marginata *from the blastoderm stage to formation and segmentation of the *regio germinalis *(A) Stage 0.** A uniform blastoderm and a region of enhanced cell density at the posterior pole of the future embryo, the so-called *cumulus*, have formed. No expression of *engrailed* (*en*) (cf. to A´). (**B**) The *regio germinalis* forms anterior to the *cumulus*; the ocular field appears as an anterior region of enhanced cell density. *en* is expressed in the future mandibular and first trunk segment (T1) (cf. to B´). (**C**) Segmentation of the *regio germinalis* begins. Transversal stripes of enhanced cell density corresponding to the later mandibular, maxillary and T1 segment appear. All anterior segments including T1 are formed from the *regio germinalis*; segments posterior to that are patterned from the segment addition zone (SAZ). At this stage the *cumulus* has transformed into the SAZ as indicated by the appearance of *en* expression corresponding to T2 in this region (cf. to C´). *en* is now expressed in the antennal and the maxillary segment (cf. to C´). (**D**) The postmaxillary segment primordium forms and expresses *en* (cf. to D´); a second stripe of *en*-expression appears in the SAZ (D´). (**E**) The premandibular segment primordium forms and expresses *en* (cf. to E´). *en* appears *de novo* in the ocular region and posterior in the SAZ (cf. to E´). The proctodaeum forms. Abbreviations: an, antennal segment; md, mandibular segment; mx, maxillary segment; oc, ocular region; P, proctodaeum; pmd, premandibular segment; pmx, postmaxillary segment; SAZ, segment addition zone; T1, first trunk segment.

We previously reported on the expression profiles of PRG orthologs in the trunk of the pill millipede, *Glomeris marginata*[24]. Here we present the expression patterns of these genes in the anterior region of the developing embryo from the blastoderm stage (stage 0) to the formation of inter-segmental grooves at approximately stage 1. Shortly after the formation of the blastoderm, a posterior zone of enhanced cell density appears (Figure 1A/A´); this domain is called the *cumulus*[11,36]. Soon after, a distinct area of enhanced cell density, the so-called *regio germinalis*, becomes visible anterior to the position of the *cumulus* and continues to grow into the anterior area of the developing embryo (Figure 1B/B´). All future anterior segments, including the first trunk segment (T1), are formed from the *regio germinalis* (Figure 1A-E). The anterior segments are patterned first but not completely simultaneously, as suggested by the expression of the SPG *engrailed* ( *en*) (Figure 1 A´-E´) [11]. The posterior segments, including T2, form sequentially from a posterior SAZ. The term “SAZ” refers to the fact that this region, from which the posterior segments are added in a one by one period, does not represent an area of enhanced cell proliferation; we therefore want to avoid the somewhat misleading terms growth- or proliferation-zone that are often used in this context (see also [37]).

Currently, it is not possible to perform mRNA detection studies (in situ hybridization) in embryos younger than stage 0. At this stage the inner vitelline membrane forms in *Glomeris*. Attempts to fix embryos at earlier developmental stages (representing development from one to six days at room temperature) in the absence of a functional vitelline membrane, have failed.

### Expression of *even-skipped* ( *eve*) in the *regio germinalis*

In *Drosophila* the *eve* gene is under control of the upstream acting maternal effect genes and gap genes, and each of the seven transversal stripes of early *eve*-expression becomes specified separately by disjoined enhancer elements (e.g. [38-40]). Because of this direct control of *eve* by the upstream level segmentation genes it represents a primary PRG. One of its important functions during early development in *Drosophila* is to indirectly regulate the segment polarity gene *engrailed* by regulating its activators *paired* and *fushi-tarazu* and its repressors *runt* and *sloppy paired*[41]. The crucial function of *eve* among the PRGs is also conserved in other insects such as *Tribolium*[20,42] and *Gryllus*[28], but may be different in other insects (e.g. [43]).

**Figure 2:**
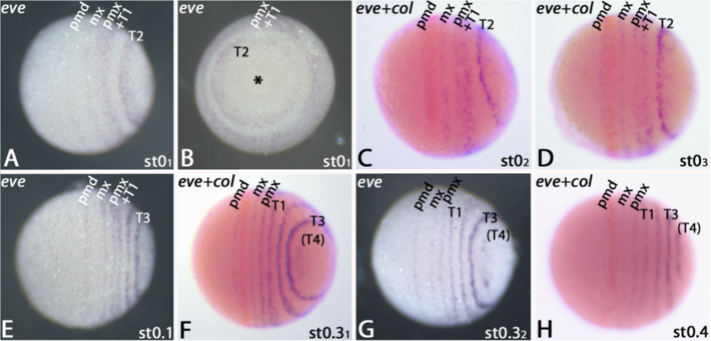
**Expression of *eve *in the *regio germinalis*.** All embryos are oriented with anterior to the left and represent ventral views, except panel (B), which represents a posterior view. Ventral (**A**) and posterior (**B**) view on an early blastoderm (stage 0_1_) embryo. Asterisk in (B) marks posterior pole of the embryo. (**C**) Later blastoderm stage embryo (stage 0_2_) double-stained for *eve* (blue signal) and *col* (orange signal). (**D**) Late blastoderm stage embryo (stage 03) double-stained for *eve* and *col*. (**E**) Stage 0.1 embryo. (**F**) Stage 0.3_1_ embryo double-stained for *eve* and *col*. Note that eve is not expressed in the mandibular segment. (**G**) Stage 0.3_2_ embryo. (**H**) Stage 0.4 embryo. The full set of anterior *eve*-stripes has appeared. Abbreviations as in Figure 1.

Expression of *Glomeris eve* is detectable at the blastoderm stage as single stripes in the future premandibular and maxillary segment, a broad domain corresponding to the postmaxillary and first trunk segment (T1), and a second broad domain located in the posterior SAZ where the future T2 is patterned (Figure 2A). The most posterior region of the embryo is free from *eve* transcripts. These cells will later sink in and form the proctodaeum. The two posterior broad expression domains are in circles around the posterior pole of the embryo (Figure 2B). The same expression is described for *eve* at later developmental stages [24,44]. The postmaxillary+T1 domain begins to resolve into two distinct stripes (Figure 2C), and the posterior domain in the SAZ transforms into a distinct stripe (Figure 2C). The embryo shown in Figure 2C is double stained for *eve* and *collier* ( *col*), which serves as a spatial landmark. At this stage the *col*-stripe is located in the posterior part of the premandibular and the anterior part of the mandibular segment [45]. It now becomes clear that the intra-segmental position of the *eve*-stripe is anterior in the premandibular segment (Figure 2C). The small gap between the posterior edge of the *col*-stripe and the stripe of *eve* expression in the maxillary segment indicates that *eve* is located anteriorly in this segment. Double staining of *eve* and the segment polarity gene *engrailed* ( *en*) supports this assumption and shows that *eve* is expressed posterior adjacent to *en*, and thus anterior in the segments (Additional file 1: Figure S1A). The intra-segmental position of *eve* in the *regio germinalis* is identical with that in the trunk segments (cf. [24]). The split of the postmaxillary+T1-domain progresses (Figure 2D), and at the subsequent stage 0.1 the split is complete (Figure 2E). A second stripe of *eve* appears in the SAZ (Figure 2E). At stage 0.3, expression corresponding to the later T4 segment appears in the SAZ. The premandibular stripe shortens, which is in accord with the changing morphology of this segment [11] (Figure 2F). The two dots that delimit the beginning of the dynamic cycles of *eve* expression in the SAZ first appear here (cf. [24]) (Figure 2G/H). Expression of *eve* in the premandibular and maxillary segments starts disappearing at stage 0.4 (Figure 2H).

### Expression of *runt (run)* in the *regio germinalis*

Like *eve*, also *run* acts as a primary PRG in *Drosophila* where one of its key functions is to regulate other PRGs as well as primary upstream acting gap genes (GGs) and maternal effect genes. In *Drosophila run* is thus an important component of the cross regulatory network of PRGs, GGs and maternal effect genes [46]. The important function of *run* in the pair rule regulatory network is conserved in short germ insects as well [20].

**Figure 3:**
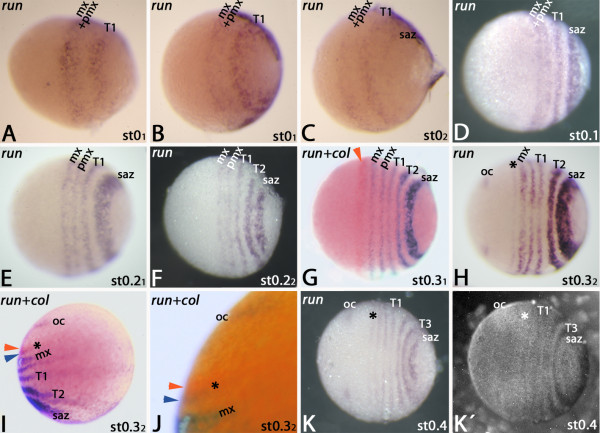
**Expression of *run *in the *regio germinalis*.** All embryos are oriented with anterior to the left and represent ventral views, except panels (I) and (J) which represent lateral views. The *run* ortholog is expressed as two broad stripes in the early blastoderm stage (**A**, **B**), of which the posterior contributes to the first trunk segment (T1) (**C**). (**D**) Stage 0.1 embryo. The anterior broad stripe splits. (**E**) Early stage 0.2 embryo. (**F**) Late stage 0.2 embryo. (**G**) Early stage 0.3 embryo double-stained for *run* (blue signal) and *col* (orange signal; expression of *col* is marked with an orange arrowhead). (**H**) Late stage 0.3 embryo. Asterisk marks faint expression in the mandibular segment. Expression in the ocular segment (oc) appears. (**I**) Late stage 0.3 embryo double-stained for *run* (expression in the mandibular segment is marked with a blue arrowhead) and *col* (orange arrowhead). Asterisk as in (H). Lateral view. (**J**) Magnification of the embryo shown in (I). Arrowheads and asterisk as in (I). (**K**) Stage 0.4 embryo. Asterisk as in (I). (**K**´) DAPI staining of the embryo shown in (K). Abbreviations as in Figure 1.

At the blastoderm stage, *run* is expressed as two broad domains corresponding to the later maxillary+postmaxillary segments and the T1 segment (Figure 3A/B). Like *everun* is also expressed in rings that surround the posterior pole of the embryo. A third ring of *run*-expression corresponding to the future T2 segment appears in the SAZ (Figure 3C), and the anterior domain begins to split into two stripes. *De novo* expression appears in the posterior SAZ as a broad domain (Figure 3D). Splitting of the maxillary+postmaxillary domain proceeds (Figure 3E/F). A stripe of *run* representing T2 has split off from the SAZ (Figure 3F). At early stage 0.3 the maxillary+postmaxillary stripe has completely split (Figure 3G) and faint expression appears in the mandibular segment posterior and adjacent to the expression of *col* (Figure 3G). Expression of *run* appears in the ocular region (Figure 3H). Co-expression of *run*+ *col* reveals that *run* is expressed posterior and adjacent to *col* (Figure 3I/J) and is thus expressed in the anterior of the mandibular segment (cf. [24]). In the dorsal extraembryonic tissue the maxillary+postmaxillary stripe is, unlike in ventral tissue, not split (Figure 3I). In stage 0.4 embryos all segmental *run*-stripes are evenly spaced; DAPI counter-staining of the same embryo reveals the position of the stripes in the anterior of the now morphologically distinguishable segments (cf. Figure 3K with K´).

### Expression of *hairy-1 (h1)* in the *regio germinalis*

In *Drosophila hairy* acts as a primary PRG [47], but this function may only be partially conserved among insects [19,20,48].

**Figure 4:**
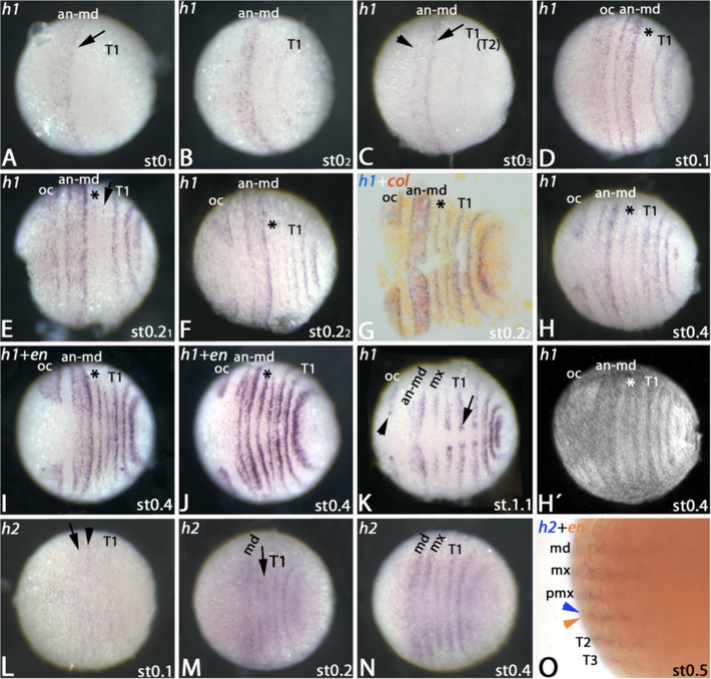
**Expression of *h1 *in the *regio germinalis*.** All embryos are oriented with anterior to the left and represent ventral views, except panel (O), which represents a lateral view. (**A**) Early blastoderm stage embryo. Arrow points to enhanced expression at the posterior rim of the anterior expression domain. (**B**) Slightly older embryo as the one shown in (A). (**C**) Late blastoderm stage embryo. Arrow as in (A). Arrowhead points to enhanced expression at the anterior rim of the broad anterior expression domain. (**D**) Stage 0.1 embryo. A second broad expression domain covering the ocular region appears. The asterisk marks very faint expression in the maxillary segment. (**E**) Early stage 0.2 embryo. Asterisk as in (D). The arrow marks faint expression in the postmaxillary segment. (**F**) Late stage 0.2 embryo. The ocular domain of *h1*-expression has split. Asterisk as in (D). (**G**) Late stage 0.2 embryo double-stained for *h1* (blue signal) and *col* (orange signal). Asterisk as in (D). (**H**) Stage 0.4 embryo. Asterisk as in (D). (**H**´) DAPI staining of the same embryo shown in (H). (**I**/**J**) Stage 0.4 embryos double-stained for *h1* and *en* (both detected as blue signals). Note that a stripe of *en*-expression lies in the broad domain of *h1*-expression covering antennal to mandibular segments. Asterisks as in (D). (**K**) Stage 1.1 stained for *h1*. Arrowhead and arrow point to enhanced expression in the developing neuroectoderm. (**L**) Expression of *h2* in a stage 0.1 embryo. Note that *h2* is not expressed at the blastoderm stage. Arrow and arrowhead mark faint expression in the mandibular and maxillary segment respectively. (**M**) Stage 0.2 embryo stained for *h2*. Arrow points to upcoming expression in the postmaxillary segment. (**N**) Expression of *h2* in a stage 0.4 embryo. (**O**) Co-expression of *h2* (blue signal) and *en* (orange signal). *h2* (blue arrowhead) is expressed anterior and adjacent to *en* (orange arrowhead). Abbreviations as in Figure 1.

In the early blastoderm, *h1* is expressed in a broad domain covering the area of the future antennal to mandibular region (Figure 4A). At the posterior rim of this domain expression is enhanced. Faint expression is visible in the tissue that will form T1 (Figure 4A/B). Expression then disappears from the centre of the antennal to mandibular domain (Figure 4C). At the same time the level of expression at the anterior rim increases resulting in two distinct stripes: one in the antennal segment and one in the mandibular segment (Figure 4C). A stripe of *h1* appears within the SAZ (Figure 4C). *De novo* expression appears in the ocular region as a broad band while expression at the posterior rim of this domain is enhanced (Figure 4D). A very faint stripe appears in the maxillary segment (Figure 4D). Then additional stripes appear simultaneously in the SAZ and in the postmaxillary segment (Figure 4E). Slightly later ventral expression of the ocular domain disappears (Figure 4F). Co-expression of *h1* with *col* reveals that the enhanced expression of *h1* in the antennal to mandibular domain lies in the posterior of the mandibular and antennal segment, respectively (Figure 4F/G). In late stage 0.4 embryos expression disappears from the ventral region of the antennal segment (Figure 4H). The *h1*-stripes that correspond to the postmaxillary segment, the maxillary segment, T1 and T2 become broadened and expression at the posterior rim becomes enhanced (Figure 4H). DAPI counter-staining reveals the affiliation of the stripes to their corresponding segments (Figure 4H´). Co-expression in similar-stage embryos stained for *h1* and *en* shows that the centre of the antennal to mandibular domain harbors the premandibular segment primordium (Figure 4I/J). The posterior rim of segmental *h1*-expression likely corresponds with the posterior portion of the developing segments (= *en* expressing tissue (cf. [11])). Enhanced dot-like expression of *h1* appears along the ventral edge of the embryo where the central nervous system forms (Figure 4K).

Expression of the second *Glomeris hairy* ortholog, *h2*, appears in transversal stripes at stage 0.1 in the mandibular, maxillary and T1 segments and weakly in the SAZ (Figure 4L). Later, expression in the postmaxillary segment appears. Expression in the mandibular and the maxillary segment broadens and expression corresponding to the T3 stripe forms in the anterior SAZ (Figure 4N). Double staining shows that *h2* is expressed anterior and adjacent to the segment polarity gene *engrailed* ( *en*) (Figure 4O and Additional file 1: Figure S1B).

### Expression of *sloppy-paired* ( *slp*) in the *regio germinalis*

In the fly *Drosophila* and the beetle *Tribolium slp* acts as a secondary PRG and is in these species regulated by the primary PRGs [20]. In *Drosophila* it acts as a gap gene in the head segments and a pair rule like regulator of SPGs in the trunk segments where it functions as an activator of *wingless* ( *wg*) and as a repressor of *engrailed* ( *en*) [8,49].

**Figure 5:**
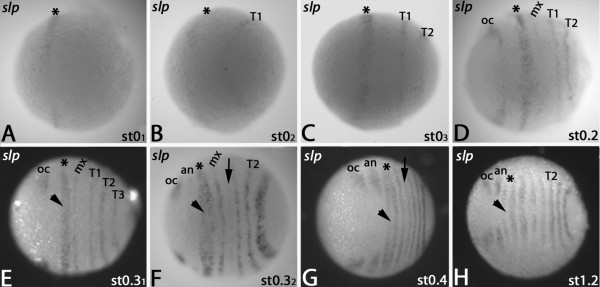
**Expression of *slp *in the *regio germinalis*.** All embryos are oriented with anterior to the left and represent ventral views. In all panels the asterisks mark progression of the early anterior expression domain. (**A**- **C**) Blastoderm stage embryos of subsequent stages. (**D**) Stage 0.2 embryo. (**E**) Stage 0.3 embryo. Arrowhead points to central region of the stripe covering premandibular+mandibular segments. (**F**) Later stage 0.3 embryo. The premandibular+mandibular stripe (asterisk) splits; transcripts in the centre of this domain disappear. The arrow points to faint expression in the postmaxillary segment. Arrowhead as in (E). (**G**) Stage 0.4 embryo. Arrow and arrowhead as in (E). (**H**) Stage 1.2 embryo. Arrowhead as in (E). Abbreviations as in Figure 1.

At the *Glomeris* blastoderm stage, *slp* is expressed as a broad domain in the future premandibular+mandibular region (Figure 5A). A stripe of *slp* appears anterior to the SAZ and corresponds to the future T1 (Figure 5B). A new domain appears in the SAZ (Figure 5C). Then *de novo* expression appears in the ocular region and faintly in the future maxillary segment (Figure 5D). Ventral expression of the former ocular stripe disappears. Expression appears in the SAZ corresponding to the future T3 segment (Figure 5E). Expression in the centre of the premandibular+mandibular domain starts disappearing and faint expression forms in the antennal region and the future postmaxillary segment (Figure 5F). Expression in the antennal and postmaxillary segment becomes stronger. Disappearing of *slp*-transcripts from the premandibular+mandibular region proceeds (Figure 5G). At stage 1.2 these stripes are completely split into two. Double staining with *engrailed* ( *en*) reveals that the segmental expression of *slp* is anterior and adjacent to *en* (Additional file Figure 1: S1C). The intrasegmental expression of *slp* is thus conserved in segments formed from the *regio germinalis* and the segment addition zone (cf. [24]). It appears thus possible that the function of *slp* as a regulator of the segment polarity genes *wg* and *en* may indeed be conserved in both, insects and myriapods.

### Expression of *pairberry-1 (pby-1)* in the *regio germinalis*

In *Drosophila* the *paired* ( *prd*) gene is classified as a so-called tertiary PRG because it functions at the lowest level of the pair rule gene cascade as a direct activator of *wingless* ( *wg*) and *engrailed* ( *en*) [50]. Expression and functional analysis of *paired* orthologs in other insects revealed that its function is conserved among insects (e.g. [51,52]).

**Figure 6:**
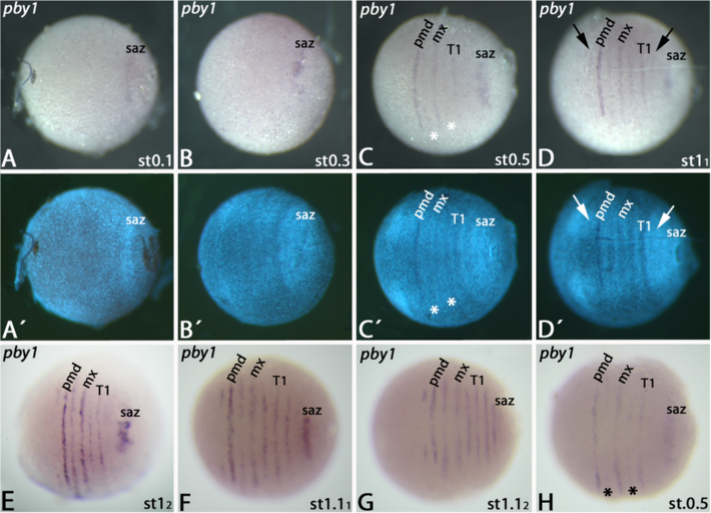
**Expression of *pby-1 *in the *regio germinalis.*** All embryos are oriented with anterior to the left and represent ventral views. (**A**) Stage 0.1 embryo. (**B**) Stage 0.3 embryo. (**C**) Stage 0.5 embryo. Segmental expression in the *regio germinalis* appears. (**D**) Early stage 1 embryo. Left arrow marks faint expression of *pby-1* in the antennal segment; right arrow marks faint expression in the second trunk segment (T2). (**E**) Late stage 1 embryo. (**F**) Early stage 1.1 embryo. (**G**) Late stage 1.1 embryo. (**H**) Stage 0.5 embryo (cf. also (C)). Critical stage in which *pby-1* is prominently transcribed in every other segment in the *regio germinalis*. Asterisks mark upcoming expression in the mandibular and postmaxillary segment. (**A**´) to (**D**´) DAPI counter-staining of the embryos shown in (A) to (D). Abbreviations as in Figure 1.

The orthologs of the *Drosophila* pax group III genes are called *pairberry*-genes because they in fact represent the orthologs of the three *Drosophila* genes *pairedgooseberry* and *gooseberry-neuro*[53]. At stage 0.1 embryos, *Glomeris pby-1* is expressed as a fuzzy domain within or directly anterior to the SAZ (Figure 6A/A´). This remains the only expression until stage 0.4 (Figure 6B/B´ and not shown). Segmental expression appears simultaneously in the premandibular, the mandibular, the maxillary, postmaxillary and the T1 segment. Expression in the premandibular, the maxillary and the T1 segment, is clearly stronger (Figure 6C, H). During stage 0.5, faint expression appears in the antennal segment and in T2. At stage 1, expression in the mandibular and the postmaxillary segment becomes stronger (Figure 6D/E). During stage 1.1, the T3-stripe first appears as a faint expression (Figure 6F) that subsequently becomes clearer (Figure 6G).

Double staining with *engrailed* ( *en*) reveals that *pby-1* is expressed anterior to *en* in anterior segments that have formed from the *regio germinalis* (Additional file 1: Figure S1D). Both genes also appear to be co-expressed in one row of cells, but this is not unambiguously clear from the available expression data (Additional file 1: Figure S1D). The intrasegmental expression of *pby-1* is conserved in anterior and posterior segments (cf. [24]), and this is consistent with a conserved regulatory function of *pby-1* in segment polarity gene regulation.

### Expression of *odd-paired* ( *opa*) and *odd-skipped* ( *odd*) in the *regio germinalis*

In *Drosophila opa* acts as a secondary PRG. An oddity of *opa* is that it is not expressed in the typical striped pattern as all the other PRGs, but it is expressed ubiquitously in the centre of the early embryo. Its presence is required but not instructive for the regulation of segment polarity genes [7,54]. In *Tribolium opa* is expessed in stripes but does not act as a pair rule gene [20].

**Figure 7:**
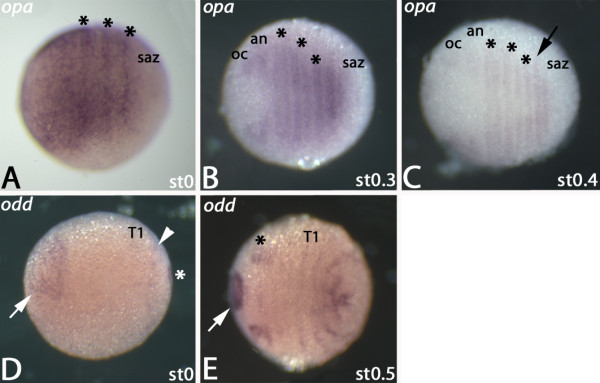
**Expression of *opa *and odd in the *regio germinalis*.** All embryos are oriented with anterior to the left and represent ventral views. (**A**) Expression of *opa* in a blastoderm stage embryo. Asterisks mark stripes of enhanced expression within a field of ubiquitous expression. (**B**) Expression of *opa* in a stage 0.3 embryo. Asterisks as in (A). Additional stripes of segmental expression form. (**C**) Stage 0.4 embryo expressing *opa*. Asterisks as in (A). The arrow points to expression in T2. (**D**) Expression of *odd* in a blastoderm stage embryo. Arrow points to expression at the very anterior of the embryo. Arrowhead points to weak expression anterior to the SAZ. Asterisk marks expression at the posterior pole of the embryo. (**E**) Stage 0.5 embryo stained for *odd* expression. Arrow as in (D). Asterisk marks dorsal expression within the gnathal segments. Abbreviations as in Figure 1.

At the blastoderm stage *opa* is ubiquitously expressed in the *regio germinalis*, but not in the SAZ (Figure 7A). Within this domain, expression is enhanced in the future premandibular+mandibular, maxillary+postmaxillary, and the T1 segment (Figure 7A). Later, the broad stripes corresponding to the premandibular+mandibular and maxillary+postmaxillary regions split. At the same time, the ubiquitous expression anterior to the premandibular segment transforms into distinct domains in the later ocular and antennal regions (Figure 7B). The T1-domain does not split. *De novo* expression corresponding to T2 appears in the SAZ (Figure 7B). Later, the T3 stripe appears in the SAZ (Figure 7C). The expression of *Glomeris opa* in a striped pattern of enhanced expression within a ubiquitous domain is thus intermediate between that of *Tribolium* (in stripes) and that of *Drosophila* (fully ubiquitous).

Interestingly, in *Drosophila* the *odd* gene is historically considered as a secondary PRG that is under control of the primary PRGs, and is repressed by *eve*[41]. In *Tribolium*, however, *odd* is part of the high-level regulatory circuit that controls secondary PRGs, and even represses *eve*[20]. Based on the find that *odd* expression is regulated through stripe specific elements, recently it has been suggested that *Drosophila odd* should rather be considered as a primary than a secondary PRG [46]. Furthermore also expression pattern analysis in another myriapod, the centipede *Strigamia*, suggests an important role for an *odd*-related gene in this species [55].

In *Glomeris* the *odd* gene is initially expressed in the most anterior area of the developing embryo, while being weakly expressed in the future T1, the SAZ and its posterior pole (Figure 7D). At stage 0.5 the anterior domain is restricted to a central position. Two patches of expression are located dorsal and posterior to this domain. The affiliation of this expression is unclear, but is possibly within future antennal, premandibular and mandibular tissue (Figure 7E). Faint expression is visible in developing segments between this domain and T1. Three stripes of expression appear posterior to T1 representing expression in the future segments T2 to T4 (Figure 7E). Altogether, the expression pattern of *odd* is not indicative for a pronounced role during the formation/patterning of segments that form from the *regio germinalis*. This implies that it does not play such a crucial role in the segmentation process in this myriapod as it does in long and short germ insects and a centipede. In segments that arise from the posterior segment addition zone (SAZ), however, *Glomeris odd* is prominently expressed in the SAZ itself and subsequently also in the dorsal segmental units [24]. This on the other hand suggests fundamental differences between the patterning of anterior vs posterior segments.

Other PRG orthologs, i.e. the paralogs *pairberry-2* ( *pby2*) and *hairy-3* ( *h3*) are not expressed in early stages in the *regio germinalis*.

## Discussion

### The *Glomeris pby-1* gene is expressed in a pattern reminiscent of that of classical PRGs

An important question that must be addressed is whether PRG orthologs may be involved in a pair rule-like mechanism during segment formation and if this is comparable to that found in the model organism, *Drosophila* (e.g.[2,18]).

**Figure 8:**
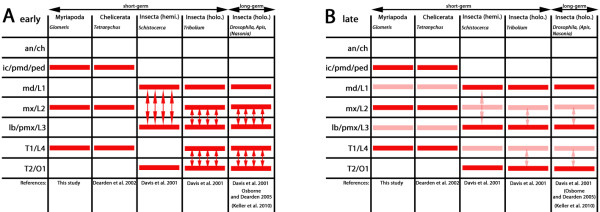
**Comparison of early *prd *ortholog expression in the blastoderm of the *myriapod Glomeris *(Myriapoda), the mite *Tetraynchus *(Chelicerata) *, and insects *(*Schistocerca, Tribolium, Nasonia, Apis *and *Drosophila*)*.*** (**A**) Early expression of *prd* orthologs in alternating segments or in double-segment wide splitting patterns. Double-headed arrows indicate for splitting expression domains. (**B**) Late segmental expression of *prd*/ *pby* orthologs. Note that expression in every other segment is enhanced or weaker, respectively. Arrows as in (A). Abbreviations: an, antennal segment, ch, cheliceral segment; hemi., hemimetabolous; holo., holometabolous; ic, intercalary segment; L1-L4, first to fourth walking leg bearing segment in chelicerates; lb, labial segment; md, mandibular segment; mx, maxillary segment; ped, pedipalpal segment; pmd, premandibular segment; pmx, postmaxillary segment; T1-T2, first and second trunk segment.

One of the investigated PRG orthologs in *Glomerispby-1*, is expressed in an early pattern in the *regio germinalis* and may be the result of an underlying classical pair rule-like mechanism. It appears simultaneously (or with very little delay) in the premandibular to T1 segment, but expression in every other segment, i.e. premandibular, maxillary and T1, is notably stronger (Figures 6 and 8). This expression profile is reminiscent of PRG expression in *Drosophila*, where an alternating pattern of weaker and stronger *prd*-stripes occurs after the splitting off of secondary stripes from primary stripes [56]. In *Glomeris pby-1*-stripes are, however, not the result of splitting. Notably, also in *Glomeris*, segmental expression of *pby-1* appears significantly later than that of the other PRGs, and even later than the segment-polarity gene *en*[11]. This late appearance of *pby-1* is in accord with its late appearance during posterior segment addition in *Glomeris*[24] and the confirmed role of *prd* as a tertiary PRG in *Drosophila*[50]. Therefore, *pby-1* cannot act at high levels in the segmentation gene cascade in *Glomeris*, and is unlikely to be involved in the regulation of the SPGs. The expression pattern of *pby-1* can however be interpreted as a possible secondary result of an underlying primary pair rule-like mechanism.

The early expression of *prd/pby* orthologs has also been examined in other insect species than *Drosophila*. In the wasp, *Nasonia vitripennis*, a long-germ insect, like *Drosophila*, stripes of *prd* expression appear in an anterior to posterior progression with every other stripe being weaker. Whether this is a result of splitting stripes like in *Drosophila* is unclear from the present data [57]. In the short-germ insect, *Tribolium*, broad stripes of *prd* expression appear that soon after split [53]. Notably, the maxillary stripe is weaker than the mandibular and labial stripes. With the elongation of the germ band, additional stripes of *prd* expression appear in the anterior of the SAZ that later split into expression in T1+T2, T3+A1 et cetera. Importantly, in *Triboliumprd* acts as a true PRG with a clear pair rule phenotype setting an example that splitting domains of double-segment wide initial expression patterns can be functionally comparable to *Drosophila* pair rule patterning [20,51]. In the hemimetabolous short-germ insect, *Schistocerca americana*, the mandibular and labial stripes of *prd* expression first appear together with a broad posterior domain that gives rise to expression in the second and third thoracic segment. With some delay weaker stripes in between (in the maxillary and the first thoracic segment) appear [3,53]. Notably, the mandibular stripe does not appear as a separate stripe but is the result of a broad splitting domain covering the gnathal arc that transforms also into the labial stripe [53].

The early pattern in long-germ and short-germ insects is therefore similar. Stripes form as broad double-segment wide domains that then split giving rise to the secondary pattern of *prd* expression (Figure 8). The result is often a secondary expression pattern with strong and weak *prd*/ *pby* expression in alternating segments (Figure 8), which is the pattern also present in *Glomeris*.

Interestingly, the same early expression profile of *prd* orthologs has also been described for the spider mite, *Tetraynchus urticae* (Chelicerata), where the paired ortholog, *Tu-pax3/7*, is initially expressed in alternating segments in the anterior body. In somewhat later stages, expression of *Tu-pax3/7* appears in the interjacent segments [30]. This pattern is virtually identical to that of *Glomeris pby-1* except the expression patterns of the first and third walking leg bearing segments (= mandibular and postmaxillary segments in *Glomeris*) in the mite are clearly delayed (Figure 8). This finding, if not caused by convergence, may place the origin of the expression pattern of *prd* orthologs (and possibly also its early function) at the very base of the Arthropoda.

The early strong expression of *Tu-pax3/7* is in the same (homologous) segments as the strong expression in *Glomeris*, but the location of the primary (stronger) *prd*/ *pby*-stripes in insects is shifted by one segment towards posterior (Figure 8). Since the homology of arthropod head segments appears to be solidly resolved by brain innervation patterns (e.g. [58,59]) and Hox gene expression patterns (e.g. [17,32,60,61]), this difference must be the result of different regulation of *prd*/ *pby* genes in the different arthropod classes.

### Expression of PRGs in double-segment wide domains: a feature of pair rule function?

**Figure 9:**
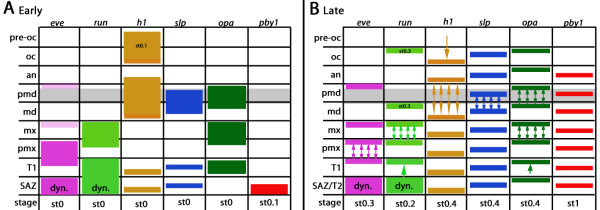
**Schematic summary of PRG expression in the *Glomeris *blastoderm.** (**A**) Early expression at the blastoderm stage. Note that the most anterior expression domain of *h1* and the expression of *pby-1* in the SAZ appear slightly later at stage 0.1. (**B**) Segmental expression at later stages. Double-headed arrows indicate splitting of initial double- (or triple-) segment wide expression domains into segmental stripes. Single-headed arrows indicate refinement of broad expression domains into a single segmental stripe of expression. Abbreviations: an, antennal segment; dyn., dynamic expression; *eve*, *even-skipped*; *h1*, *hairy-1*; md, mandibular segment; mx, maxillary segment; oc, ocular field; *opa*, *odd-paired*; *pby-1*, *pairberry-1*; pmd, premandibular segment; pmx, postmaxillary segment; pre-oc, pre-ocular region; *run*, *runt*; SAZ, segment addition zone; *slp*, *sloppy paired*; st, stage; T1-T2, first and second trunk segment. The grey bar indicates expression of the molecular landmark collier.

We find that in *Glomeris* all PRGs except *pby-1* are initially expressed in double- or multiple-segment wide domains in at least some segmental primordia (Figure 9). Most of the broad expression patterns in *Glomeris* extend into two adjacent future segments (Figure 9). Splitting of double-segment wide expression domains of PRGs is found also for one of the *Drosophila* PRGs, namely *paired* ( *prd*) [53], and for a number of PRGs in other insects (e.g. [23,52,53]) (Figure 8) (discussed above). Functional studies in the beetle, *Tribolium*, have shown that this kind of expression pattern is indeed connected to classical pair rule phenotypes [19], but [20].

It is possible that the splitting of double-segment wide expression domains is an ancestral regulatory feature of arthropod PRGs, because it is present in the blastoderm of insects, a myriapod (this study) and also a spider [31].

Initial expression of PRGs in broad domains may be a genetic constraint, because their early expression patterns are likely to be regulated by the gap-genes (GGs), as known from insects (e.g. [23,62-65]). Since the GGs are expressed in broad domains they may activate PRGs that are also initially located in broad domains, but that then transform into segmental stripes, possibly by the combinatorial action of the PRGs themselves.

### Blastoderm patterning in long- and short-germ arthropods

In the model arthropod, *Drosophila melanogaster*, all segments are patterned at the blastoderm stage. This, however, represents a derived developmental mode, and hence the segmentation gene cascade known to act in *Drosophila* cannot function in the same way in short-germ arthropods that add posterior segments sequentially from a posterior SAZ (e.g. [66]).

Functional studies and gene expression analysis have shown that the PRGs are likely to be involved in segment formation in non-insect arthropods (e.g. [24-26,55,67,68]). Despite that, it was largely unclear whether PRGs are also involved in anterior patterning in non-insect arthropods, as only very few studies examine PRG function and/or expression at early blastoderm stages in non-insect arthropods [30,31].

The data presented here suggest that most of the investigated PRGs in *Glomeris* are involved in segmental patterning of the blastoderm. All PRGs (except *odd-skipped*) are expressed in transversal stripes corresponding to one or multiple segment primordia (discussed above). Expression of any given PRG does not appear simultaneously or in an anterior posterior order, but with minimal temporal variance in different segmental primordia. Furthermore, the order of appearance of the segmental primordia differs for every PRG ortholog (Figure 9). This is comparable to what happens in *Drosophila*, where the PRGs often appear in an irregular progression in the blastoderm and the initial expression is often in broad domains and not in the classical seven-stripe pattern (e.g. [54,56,69]).

The stereotypic appearance of the PRGs in the *regio germinalis* in *Glomeris* is superficially reflected by the appearance of the SPG *en*[11]. *en* transcription starts later compared to when most of the PRGs are transcribed, which is in accord with a possible regulatory function of some of the PRGs on *en* in the anterior embryo in *Glomeris*. While the PRGs appear to be active before the onset of the SPGs [11] and the expression of the Hox genes [32], the anterior acting GGs are expressed as early as, or possibly earlier [70].

The principal hierarchy of segmentation gene interaction known from *Drosophila* with GGs regulating PRGs and PRGs regulating SPGs can be conserved in *Glomeris* as well, at least with respect to segment formation in the blastoderm.

It is tempting to speculate that anterior patterning is indeed conserved among long- and short-germ arthropods and that this possibly ancestral patterning mechanism has been extended to the complete embryo in *Drosophila* and other long-germ insects. Results of this transition may have been the recruitment of the posterior acting GGs and the loss of the posterior segmentation clock as suggested by [71].

### Pair rule-like mechanism in posterior segment addition?

Patterning of segments in pairs may be an ancestral mechanism (discussed above). The dynamic expression of some PRGs in the posterior SAZ in myriapods [14,24,55,68] may be the equivalent of double-segment wide stripes of PRGs in the blastoderm. This condition is most evident in the centipede, *Strigamia*, where the addition of posterior segments occurs in pairs and with the involvement of PRGs, such as *even-skipped*, from the posterior SAZ [68]. Further evidence for this hypothesis comes from centipedes where the number of trunk segments is always odd (reviewed in [72,73]). This shows that there may be a genetic constraint that does not allow for the formation of an even number of trunk segments in centipedes [74].

Furthermore, in *Glomeris* the number of trunk segments is always 17 for females and 19 for males. This indicates that the posterior segmentation clock in *Glomeris* males may produce another two segments by adding one cycle of dynamic gene expression during its development.

## Conclusions

We have found evidence, in the form of gene expression patterns, that *Drosophila* pair rule gene orthologs are also likely involved in anterior body patterning in the myriapod *Glomeris marginata*. This finding, however, requires further investigation through functional studies, which, at the moment, have not yet been established for *Glomeris*, or any other myriapod species. The expression patterns found in *Glomeris* are, to some extent, similar, and thus reminiscent of true pair rule patterning as seen in *Drosophila*. Comprehensive comparative expression data from other arthropods, and especially crustaceans, are necessary to gain a better understanding of the ancestral mode(s) of arthropod segmentation.

## Authors´ contributions

RJ designed the study, conducted the experiments and wrote the first draft manuscript. WGMD and GEB were involved in data discussion and writing the final version of the manuscript. All authors approved the final version of the manuscript.

## Supplementary Material

Additional file 1 **Figure S1.** Intrasegmental expression of PRGs revealed by double-staining with the SPG engrailed (en). In all panels anterior is to the left. A Stage 1.2 embryo, flat-mounted. Double staining of *en* (orange signal) and *even-skipped* ( *eve*, blue signal). B Stage 0.5 embryo, whole mount. Double staining of *en* (orange signal) with *hairy-2* ( *h2*, blue signal). C Stage 0.5 embryo, flat-mounted. Double staining of en (orange signal) and *sloppy-paired* (blue signal). Note that the anterior of the germ band was damaged during the process of mounting and removing the yolk. D Stage 1.1 embryo, flat-mounted. Double staining of *en* (orange signal) and *pairberry-1* (blue signal). Abbreviations: an, antennal segment; md, mandibular segment; pmd, premandibular segment; pmx, postmaxillary segment; T1 and T3, first and third trunk segment. Click here for file
