# Supplementary figures and images for: Expression of pair rule gene orthologs in the blastoderm of a myriapod: evidence for pair rule-like mechanisms?
Source: BMC Dev Biol. 2012 May 17;12:15. doi: 10.1186/1471-213X-12-15 (PMC3477074; doi:10.1186/1471-213X-12-15)

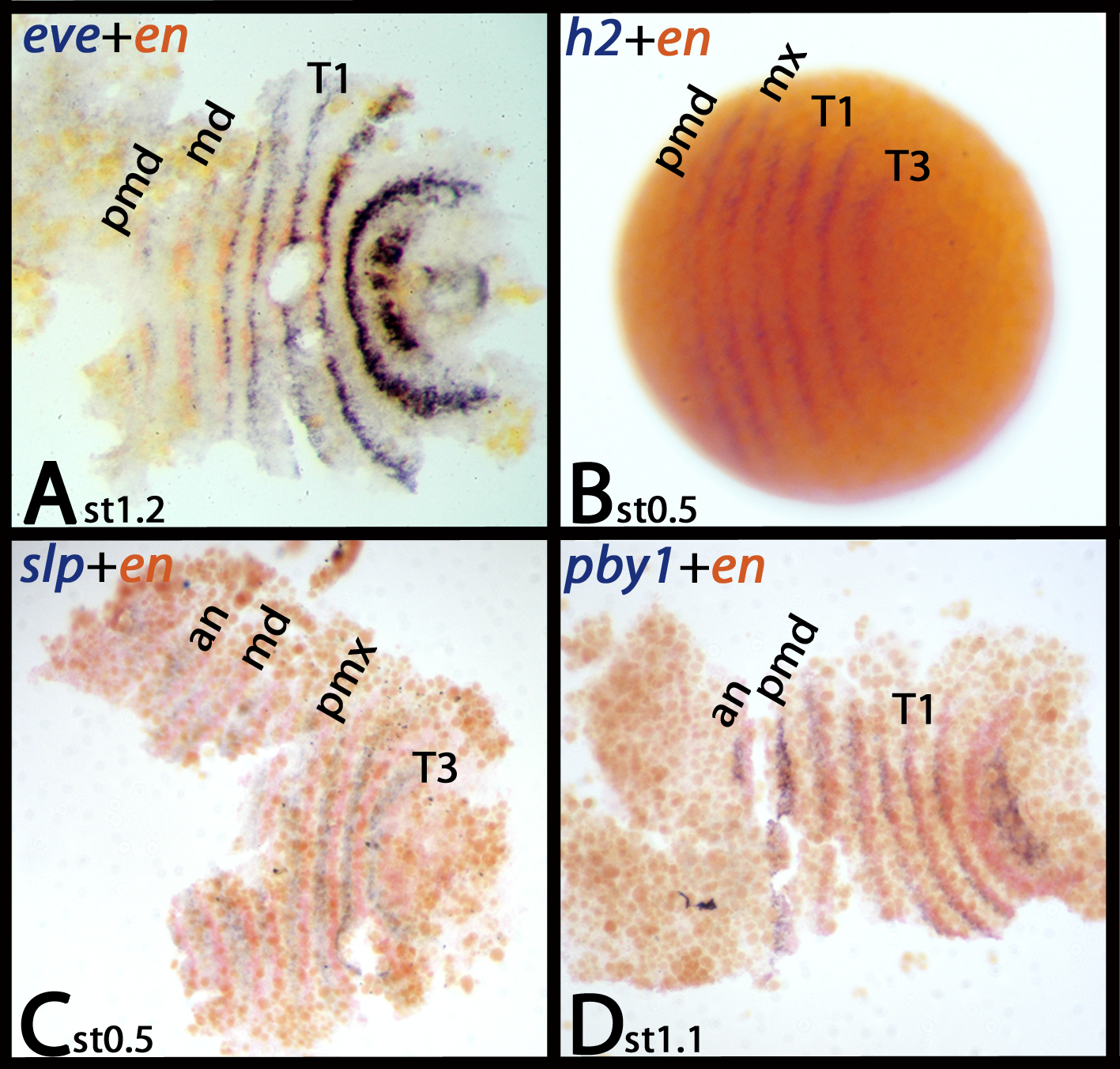

Supplement: Additional file 1 — Figure S1. Intrasegmental expression of PRGs revealed by double-staining with the SPG engrailed (en). In all panels anterior is to the left. A Stage 1.2 embryo, flat-mounted. Double staining of en (orange signal) and even-skipped ( eve, blue signal). B Stage 0.5 embryo, whole mount. Double staining of en (orange signal) with hairy-2 ( h2, blue signal). C Stage 0.5 embryo, flat-mounted. Double staining of en (orange signal) and sloppy-paired (blue signal). Note that the anterior of the germ band was damaged during the process of mounting and removing the yolk. D Stage 1.1 embryo, flat-mounted. Double staining of en (orange signal) and pairberry-1 (blue signal). Abbreviations: an, antennal segment; md, mandibular segment; pmd, premandibular segment; pmx, postmaxillary segment; T1 and T3, first and third trunk segment. [file 1471-213X-12-15-S1.tiff]
